# Round the Hospitals

**Published:** 1916-11-04

**Authors:** 


### ROUND THE HOSPITALS.

At a meeting of the Central Committee for the State Registration of Nurses held on October 21 a resolution was passed rejecting Mr. Stanley's efforts to arrive at an agreed Bill, and stating that the Committee had determined to proceed with its own Registration Bill. The constitution of the Nursing Council appears to be the rock on which the Committee and the College have split, the former refusing to agree to the clause providing that the first Council shall consist of forty-five persons whose names are to be inserted in the Bill. As we have already pointed out, the inclusion of the names in the Bill is the surest guarantee that the first Council, which will be in power for the next two years, will be representative and trustworthy. The first two years will be years of crisis and difficulty, and it is essential that during this period those who have charge of the affairs of the College and of the Register shall be trusted workers in the nursing world. In no other way can this end be secured than by the inclusion of their names in the Bill, and the College of Nursing, backed by the leaders of the nursing profession, is proceeding with its Bill on these lines.

---

The high esteem in which Miss M. S. Donaldson, the late matron of Mount Vernon Hospital, Northwood, was held was evidenced at the recent meeting of the committee, when she was presented by the Chairman, Mr. C. Johnston, with a substantial cheque as a mark of the committee's unanimous appreciation. Mr. Johnston laid stress on the value of Miss Donaldson's work and the high standard of character and knowledge which she has inculcated during the eleven years of her matronship in the nurses who have trained under her. The nursing and domestic staff on the same occasion paid tribute to their late matron, and expressed in words and by gift their gratitude for her labours on their behalf. In addition to the cheque the committee propose to present Miss Donaldson with a memento of their pleasant association with her in the work of the hospital. The nursing world will be glad to hear that Miss Donaldson is not retiring from work in the field to which she has devoted so much of her life, but is looking forward to taking a more prominent part than hitherto in public work for her profession.

A correspondent writes to complain of the numerous injustices and inequalities in regard to pay and promotion of nurses in the British Expeditionary Force in France. She says that some nurses receive promotion to the rank of sister after comparatively short periods of service—often home service—whereas others work hard for years abroad without receiving similar recognition. It is quite probable that many inequalities of this sort exist in the Q.A.I.M.N.S. and in its auxiliary Reserve and Territorial branches. It is also quite probable that some of these inequalities arise from want of judgment and discrimination on the part of the matrons and principal matrons, upon whose recommendation such promotion must in the nature of things depend. The currying of favour by the children of this world is not unknown in civil hospital nursing; and feminine nature is not changed by putting it into grey and scarlet. But most of the topsy-turvydom of which our correspondent complains is due, in our opinion, to the inevitable drawbacks of unpreparedness. Throughout the Army, in all its branches, the more competent can be found serving under the orders of the less competent; and there is no way of avoiding this kind of injustice in a scratch Army hastily assembled to meet an emergency such as that which confronts the British Empire to-day. The remedy is preparedness; perhaps when nurses get votes they will so bestow them that statesmen capable of thinking of the nation's welfare, instead of party hacks, shall be placed in power.

Readers of The Hospital who have followed the correspondence and articles which appeared in its columns in August and September on the National Poor-Law Officers' Association, concluding with the article in its issue of September 9, p. 547, entitled "Why It Cannot Represent Trained Nurses," will be interested to learn that at the annual meeting held last week of the Poor-Law Infirmary Matrons' Association the question was discussed as to whether the members of the latter Association considered that it would be an advantage for nurses trained or in training to join the National Poor-Law Officers' Association. The President of the Poor-Law Infirmary Matrons' Association pointed out that as an Association they could not influence the action of individual members, but it was desired they should express their opinion as a corporate body on the subject. When the vote was taken, the opinion of those present at the meeting proved to be unanimously against the suggestion that it was in any way advantageous for nurses trained or in training to join the National Poor-Law Officers' Association, while of those absent six of the members were in favour of the suggestion while twelve were against it.

Throughout the country district nursing associations are suffering from increase of work involved owing to members of their staffs being called up for military duty, and the difficulty or impossibility of replacing them. At Liverpool, the Queen Victoria District Nursing Association are, in consequence of this shortage, willing to take nurses with a shorter training than has hitherto been regarded as essential, but, notwithstanding this step, they have had to intimate to the Education Committee that they will not be able to supply nurses to attend to the minor ailments of the elementary school children. Unless more financial help is received a further curtailment of the work is foreshadowed in the report for 1915. The salaries of matrons and nurses at the seven homes comprised in the Association are to be increased, and the ability to maintain a sufficient staff for the proper performance of the work is made dependent on the amount of contributions which may be received. Seeing that the work of nursing the sick poor in their own homes originated in Liverpool in 1859, and that the scheme was extended and welded into one system in 1898, when the Liverpool Association was founded in commemoration of Queen Victoria's reign and affiliated to the Queen Victoria Jubilee Institute, it is to be hoped that Liverpool will come forward handsomely to prevent the curtailment of its useful and necessary work.

A fortnight ago (The Hospital, October 21, 1916, p. 53) we announced the formation of a new Institute of Massage at Manchester. The long-established London Society—namely, The Incorporated Society of Trained Masseuses—has issued a letter drawing attention to the fact that the new Manchester organisation is not connected with it in any way, and adverting to the disadvantage of destroying the "one-portal" system, which has hitherto existed in the profession of skilled and reputable massage. The letter also states that when the Manchester scheme was mooted, the Incorporated Society endeavoured to arrange a conference so as to avoid disunion and division, but that the offer was rejected. Schisms of this sort are, in general, to be deprecated. They seldom make for strength; but are sometimes inevitable. More can scarcely be said usefully at present.

With the exception of the seven nurse members, the first Scottish Board of the College of Nursing is now complete, and at its first meeting, to be held before our next issue is published, it will proceed to the business of electing a chairman and secretary, the selection of an office and the co-optation of the nurse-members. It is understood that Miss Gill, lady superintendent of Edinburgh Royal Infirmary, has been asked to undertake the direction of the secretariat. Her acceptance of this office would be a source of strength to the Board, and the nursing profession as a whole.

For Matrons' and Sisters' Appointments, see p. xviii; For The District Nurse, see p. 107.

